# Supplementary material for: Hospital Incidence, Sex Disparities, and Perioperative Mortality in Open Surgically Treated Patients with Aneurysms of the Ascending Aorta and Aortic Arch in Switzerland
Source: Healthcare (Basel). 2024 Feb 2;12(3):388. doi: 10.3390/healthcare12030388 (PMC10855317; doi:10.3390/healthcare12030388)
Supplement: Supplementary file 1 [file healthcare-12-00388-s001.zip › healthcare-2778927-supplementary/Table S1.pdf]

Table S1. Utilised codes of procedures (CHOPS) and diagnoses (ICD-10)

**Definitions**	
**Thoracic Aneurysm**	ICD-10: I71.1, I71.2<br>*AND*<br>CHOP-2018-Aorta: 38.45.00, 38.45.10, 38.45.11, 38.45.12, 38.45.14, 38.45.19, 38.45.20, 38.45.29, 38.64, 38.40, 38.52, 38.45<br>*AND*<br>CHOP-2018-ECC: 39.61.00, 39.61.1, 39.61.10, 39.61.11, 39.61.12, 39.61.13, 39.61.14, 39.61.15, 39.61.2, 39.61.21, 39.61.22, 39.61.23, 39.61.24, 39.61.25, 39.61.26, 39.61.99
**ICD-10 main Diagnosis (Details):**	
I71.1	Aneurysma der Aorta thoracica, rupturiert
I71.2	Aneurysma der Aorta thoracica, ohne Angabe einer R uptur
**CHOP-2018-Aorta (Details):**	
38.45.00	Resektion von thorakalen Blutgefässen mit Ersatz,n.n.bez.
38.45.10	Resektion der thorakalen Aorta mit Ersatz,n.n.bez.
38.45.11	Resektion der Aorta ascendens oder des Aortenbogens mit Ersatz
38.45.12	Resektion der Aorta thoracica mit Ersatz
38.45.14	Available since 2011: Resektion der thorakalen Aorta und Ersatz mit Hybridprothese
38.45.19	Resektion der thorakalen Aorta mit Ersatz, sonstige
38.45.20	Resektion sonstiger thorakaler Arterien mit Ersatz, n.n.bez.
38.45.29	Resektion sonstiger thorakaler Arterien mit Ersatz
38.64	Only available in 2009/2010: "Sonstige Exzision der Aorta (bogen, ascendens, descedens, abdominalis)"
38.40'	Only available in 2009/2010: "Resektion von Blutgefässen mit Ersatz, Lokalisation n.n.bez."
39.52	Only available in 2009/2010: "Aneurysmorrhaphie durch oder mit Anastomose Aorta thoracalis"
38.45	Only available in 2009/2010: Resektion von thorakalen Blutgefässen mit Ersatz
**CHOP-2018-ECC (Details):**	
39.61.00	Extrakorporelle Zirkulation (ECC), n.n.bez.
39.61.1	Konventionelle extrakorporelle Zirkulation Herz-Lungen-Maschine
39.61.10	Konventionelle extrakorporelle Zirkulation (ECC) unter Normothermie (mehr als 35 °C)

39.61.11	Konventionelle extrakorporelle Zirkulation (ECC) unter milder Hypothermie (32 - 35 °C)
39.61.12	Konventionelle extrakorporelle Zirkulation (ECC) unter moderater Hypothermie (26 bis unter 32 °C)
39.61.13	Konventionelle extrakorporelle Zirkulation (ECC) unter tiefer Hypothermie (20 bis unter 26 °C)
39.61.14	Konventionelle extrakorporelle Zirkulation (ECC) unter profunder Hypothermie (unter 20 °C)
39.61.15	Konventionelle extrakorporale Zirkulation (ECC) unter Hypothermie, unbekannte Temperatur
39.61.2	Minimalisierte extrakorporelle Zirkulation
39.61.21	Minimalisierte extrakorporelle Zirkulation unter Normothermie (mehr als 35 °C)
39.61.22	Minimalisierte extrakorporelle Zirkulation unter milder Hypothermie (32 - 35 °C)
39.61.23	Minimalisierte extrakorporelle Zirkulation unter moderater Hypothermie (26 bis unter 32 °C)
39.61.24	Minimalisierte extrakorporelle Zirkulation unter tiefer Hypothermie (20 bis unter 26 °C)
39.61.25	Minimalisierte extrakorporelle Zirkulation unter profunder Hypothermie (unter 20 °C)
39.61.26	Minimalisierte extrakorporelle Zirkulation unter Hypothermie, unbekannte Temperatur
39.61.99	Extrakorporelle Zirkulation (ECC), sonstige

CHOP-2018-Aortenklappenersatz (Details):

35.F1	Aortenklappenersatz durch Herzklappenprothese oder klappentragende Gefässprothese oder klappentragende Gefässprothese
35.F1.0	Detail der Subkategorie 35.F1
35.F1.00	Aortenklappenersatz durch Herzklappenprothese oder klappentragende Gefässprothese, n.n.bez.
35.F1.09	Aortenklappenersatz durch Herzklappenprothese oder klappentragende Gefässprothese, sonstige
35.F1.1	Aortenklappenersatz durch Allograft (Homograft)
35.F1.11	Aortenklappenersatz durch Allograft (Homograft), über vollständige Sternotomie
35.F1.12	Aortenklappenersatz durch Allograft (Homograft), über minimal-invasive Thorakotomie (partielle obere Sternotomie, laterale Thorakotomie)
35.F1.2	Aortenklappenersatz durch Xenograft
35.F1.21	Aortenklappenersatz durch Xenograft, über vollständige Sternotomie
35.F1.22	Aortenklappenersatz durch Xenograft, über minimal-invasive Thorakotomie (partielle obere Sternotomie, laterale Thorakotomie)
35.F1.23	Aortenklappenersatz durch Xenograft, endovaskulärer Zugang
35.F1.24	Aortenklappenersatz durch Xenograft, transapikal

35.F1.25	Aortenklappenersatz durch Xenograft, transapikal perkutan
35.F1.3	Aortenklappenersatz durch Xenograft, stentless
35.F1.31	Aortenklappenersatz durch Xenograft, stentless über vollständige Sternotomie
35.F1.32	Aortenklappenersatz durch Xenograft, stentless über minimal-invasive Thorakotomie (partielle obere Sternotomie, laterale Thorakotomie)
35.F1.33	Aortenklappenersatz durch Xenograft, stentless endovaskulärer Zugang
35.F1.34	Aortenklappenersatz durch Xenograft, stentless transapikal
35.F1.35	Aortenklappenersatz durch Xenograft, stentless transapikal perkutan
35.F1.4	Aortenklappenersatz durch Xenograft, nahtfrei
35.F1.41	Aortenklappenersatz durch Xenograft, nahtfrei über vollständige Sternotomie
35.F1.42	Aortenklappenersatz durch Xenograft, nahtfrei über minimal-invasive Thorakotomie (partielle obere Sternotomie, laterale Thorakotomie)
35.F1.43	Aortenklappenersatz durch Xenograft, nahtfrei endovaskulärer Zugang
35.F1.44	Aortenklappenersatz durch Xenograft, nahtfrei transapikal
35.F1.45	Aortenklappenersatz durch Xenograft, nahtfrei transapikal perkutan
35.F1.5	Aortenklappenersatz durch mechanische Prothese
35.F1.51	Aortenklappenersatz durch mechanische Prothese, über vollständige Sternotomie
35.F1.52	Aortenklappenersatz durch mechanische Prothese, über minimal-invasive Thorakotomie (partielle obere Sternotomie, laterale Thorakotomie)
35.F1.6	Aortenklappenersatz durch klappentragende Gefässprothese, biologisch (incl. Composite Graft)
35.F1.61	Aortenklappenersatz durch klappentragende Gefässprothese, biologisch, über vollständige Sternotomie
35.F1.62	Aortenklappenersatz durch klappentragende Gefässprothese, biologisch, über minimal-invasive Thorakotomie (partielle obere Sternotomie, laterale Thorakotomie)
35.F1.7	Aortenklappenersatz durch klappentragende Gefässprothese, mechanisch (incl. Composite Graft)
35.F1.71	Aortenklappenersatz durch klappentragende Gefässprothese, mechanisch, über vollständige Sternotomie
35.F1.72	Aortenklappenersatz durch klappentragende Gefässprothese, mechanisch, über minimal-invasive Thorakotomie (partielle obere Sternotomie, laterale Thorakotomie)

**ICD-10 Comorbidities:**	
Literature	Elixhauser Comorbidity Score (ES)
I25*	Chronic ischemic heart disease
I50*	Chronic heart failure
G45*, G46*, H340*, I6*	Cerebrovascular disease
Elixhauser Item No. 6	Hypertension
Elixhauser Item No. 9	Chronic pulmonary disease
Elixhauser Item No. 10 and 11	Diabetes mellitus
Elixhauser Item No. 13	Chronic kidney disease
Elixhauser Item No. 17, 18 and 19	Cancer
Elixhauser Item No. 22	Obesity
Q87.4	Marfan-Syndrome
**ICD-10 Perioperative Diagnosis / Complications:**	
I21.*, I22.*	Acute/Recurrent myocardial infarction
I63.4	Acute stroke
G95.1	Acute paraplegia (incl. spinal infarction)
I74.*	Acute limb ischemia
K55.0	Acute mesenteric infarction
N28.0	Acute renal artery infarction
