# Supplementary material for: Hospital Incidence, Sex Disparities, and Perioperative Mortality in Open Surgically Treated Patients with Aneurysms of the Ascending Aorta and Aortic Arch in Switzerland
Source: Healthcare (Basel). 2024 Feb 2;12(3):388. doi: 10.3390/healthcare12030388 (PMC10855317; doi:10.3390/healthcare12030388)
Supplement: Supplementary file 1 [file healthcare-12-00388-s001.zip › healthcare-2778927-supplementary/Table S2.pdf]

Table S2. Treatment and Outcome of palliatively treated Ruptured Ascending and Arch Aortic Aneurysms

	male (N=79)	female (N=75)	Total (N=154)	p value
**Destination after discharge**				0.526
Home	3 (3.8%)	4 (5.3%)	7 (4.5%)	
Other	0 (0.0%)	1 (1.3%)	1 (0.6%)	
Deseased	76 (96.2%)	70 (93.3%)	146 (94.8%)	
**Hospital Mortality**	76 (96.2%)	70 (93.3%)	146 (94.8%)	0.423
